# Phosphate Low-Melting Glasses as Synergist in Flame-Retardant Cable Sheath Composition: Performance and Mode of Action

**DOI:** 10.3390/polym17192679

**Published:** 2025-10-03

**Authors:** Diana Amin Alsayed, Rodolphe Sonnier, Belkacem Otazaghine, Patrick Jean, Yves Brocheton, Laurent Ferry

**Affiliations:** 1 Polymers Composites and Hybrids (PCH), IMT Mines Ales, 30100 Ales, France; diana.amin-alsayed@mines-ales.fr (D.A.A.); rodolphe.sonnier@mines-ales.fr (R.S.); belkacem.otazaghine@mines-ales.fr (B.O.); 2 Corning European Technology Center, Corning SAS, 7 bis Avenue de Valvins, 77210 Avon, France; jeanp@corning.com (P.J.); brochetoy@corning.com (Y.B.)

**Keywords:** flame retardant, low-melting glass, ATH, polyethylene, cable

## Abstract

Nowadays, fiber optic cables are a strategic issue because of their importance in telecommunications. Due to the densification of optic cables and the reduction in polymeric layer thickness, the flammability of the external sheath has to be improved. Three novel flame-retardant compositions using phosphate low-melting glasses (LMGs) as aluminum trihydrate (ATH) synergist were assessed in a polyethylene–ethylene vinyl acetate (PE-EVA) matrix. It was highlighted that LMG at a 10 wt% content reduced the peak and mean value of heat release rate (HRR), respectively, to 142 and 90 kW/m^2^ corresponding to 52% and 42% reduction compared to ATH only. Potassium phosphate LMG was shown to perform better than sodium or zinc phosphate LMG. The improvement was assigned to the formation of an expanded mineral layer at the surface of the material during combustion that acts as a thermal shield slowing down the pyrolysis rate. The structural analysis revealed that the presence of alkaline cations in glasses led to short phosphate chains that resulted in low softening point and low-viscosity liquid. It was evidenced that under heat exposure the melted glass is likely to flow between the dehydrating ATH particles, creating a cohesive layer that expands. Additionally, interactions between ATH and LMG were also evidenced. The new crystalline species may also play a role in the cohesion of the layer.

## 1. Introduction

Optical fibers have changed modern industries because they are the backbone of high-speed communications and data networking [1]. Optical cables are assemblies that enable the gathering and protection of a great number of optical fibers. Like other cables, optical cables have to fulfill various specifications including those related to their reaction to fire. In this respect, cable sheath composition is of paramount importance. The most common composition for the outer layer of cable includes polyolefin as matrix and hydrated mineral fillers as the flame retardant [2,3]. Due to the high flammability of polyolefins, a high amount of fillers is required to obtain good fire performance (up to 65 wt%) [4]. According to Hull et al. [5], who investigated the mode of action of ATH using thermal analysis and the literature data, 55% of the fire performance effect would be due to the endothermal decomposition of the mineral filler, 23% to the effect of water vapor in gaseous phase, 13% ascribed to the formation of a barrier layer, and 9% to the absorption of heat by the filler. It can be observed that the contribution of the barrier layer formed by the remaining dehydrated alumina is relatively low due to its low mechanical cohesion [6]. Various efforts have been made to improve the cohesion of the residual mineral layer by replacing a part of the hydrated fillers with other compounds [7].

In this respect, many studies over the last twenty years involved the use of nanoparticles as synergists of ATH. The most studied nanoparticles were probably organo-modified layered silicates, especially montmorillonite. Their modes of action in polyolefins have been evidenced by various authors and involve both physical and chemical mechanisms [8,9,10,11,12]. Physical ones correspond to the building of a barrier layer including the lamellar particles and eventually the expansion [13,14,15] and modification of heat and mass transfer, as well as the modification of polymer chain mobility. Chemical mechanisms are related to the catalytic effect of layered silicates on the degradation pathway of polymers likely to promote the formation of char. Silica or silica-based compounds represent another group of synergists that can be used in combination with hydrated fillers. Silica particles do not necessarily strongly modify the pHRR value but generally play a role in the structuration of the barrier layer and may prevent its fracture. Moreover, silica particles do modify the viscosity of the degrading polymer which may also promote the expansion of the protective layer [16,17,18].

Another strategy to improve the fire behavior of polymers consists of creating a mineral barrier using low-melting glass systems. The barrier acts as a thermal shield that protects the underlying material. The pioneer works in this field are those performed by BF Goodrich in the 80s. Kroenke studied low-melting sulfate glasses and glass ceramics as fire retardants and smoke suppressants in PVC [19]. The glass composition was based on K_2_SO_4_/Na_2_SO_4_/ZnSO_4_ ternary mixture. The compositions could be improved by adding transition metals such Ni, Cu, Mn, or V. Low-melting glasses and ceramics promote the formation of an intumescent layer and also reduce the production of smoke. Based on the method of Kroenke, Myers and Licursi developed a sulfate–phosphate glass and compared its effect to a borate–carbonate glass and ammonium pentaborate [20]. The fire behavior was characterized by a torch test that assessed the thermal stability and the insulative properties of the residual layer formed during combustion. The glasses were used in rigid and plasticized PVC. The results showed that the protection effect is particularly spectacular when glass formation is accompanied by intumescence. Low-melting sulfate–phosphate glasses were patented by Goodrich as an effective intumescent flame retardant for polymers [21]. Myers et al. dedicated another study to ammonium pentaborate (APB) as a flame retardant in thermoplastic polyurethane. It was shown that the glassy intumescent structure promoted by APB during combustion serves as an effective fireproofing system [22].

Later, in the 90s, a series of boron-containing compounds likely to act as flame retardants by the formation of a vitreous protective layer during burning were developed. The main products were 2ZnO, 3B_2_O_3_, 3.5H_2_O and 4ZnO, B_2_O_3_, H_2_O. These compounds are also hydrated products that may act by the endothermic release of water. Depending on the composition, water is released between 290 and 415 °C. Zinc borates (ZB) were used in combination with hydrated mineral fillers (ATH or MDH) where they play the role of sintering aid that promotes the formation of a strong char/ceramic residue [23]. They were highlighted to also be smoke suppressants. Bourbigot et al. evidenced that the combination of ATH/ZB or MDH/ZB in EVA led to a strong decrease in pHRR in cone calorimeter and an increase in LOI of up to 52% [24,25]. ZB was also studied in combination with MDH and talc. It was highlighted that these combinations enabled the increase in LOI of up to 56.4% and obtained V0 rating in UL94 [26]. In 2000, a fire barrier material was patented by Wolstenholme International Limited [27]. This material is a combination of two frits: one low-melting frit (450 °C) and one high-melting frit (700 °C). The composition includes zinc borate, magnesium carbonate, zinc carbonate, and sodium pyrophosphate. The patent claimed that this material is particularly suitable for thermoplastics. Wu et al. used Ceepree glass in epoxy resin in combination with organo-modified MMT [28]. The best results were obtained with pure Ceepree with a drastic decrease in pHRR and the formation of a cohesive char at the sample surface.

Yu et al. synthesized low-melting glass from silane precursors [29,30,31]. After grinding, this glass was used alone or combined with o-MMT in an epoxy resin. The combination of layered silicate with glass enabled the formation of an efficient protective layer and drastically reduced the HRR. More recently Liu et al. synthesized phosphate-based low-melting glasses containing sodium and calcium oxides as modifiers [32]. The glass transition temperature ranged from 260 °C to 410 °C. Some of these glasses were used as synergists of ammonium polyphosphate (APP) in flame-retardant epoxy composition. Great improvement in fire performance was observed for the composition containing 9% APP and 1% of glass with V0 ranking at UL94 and a significant decrease in the peak of heat release rate and smoke production [33].

This work deals with three different types of low-melting-point phosphate glasses (LMG1, LMG2, and LMG3) used as a synergist in flame-retardant compositions. The novelty of the work lies in the chemical compositions of LMGs that enable lower glass transition temperatures compared to the literature, thus triggering a faster formation of the protective barrier layer. These LMGs were used for the first time in PE/EVA/ATH compositions for optical cable sheath applications. A series of comprehensive fire tests and advanced characterization methods were carried out to evaluate the performance and structure of materials with the final goal of supplying a comprehensive view of the modes of action of the flame-retardant system.

## 2. Materials and Methods

### 2.1. Materials

The polymer matrix utilized in this study comprises ethylene vinyl acetate (EVA), tradename EVATANE 24-03 from Arkema (Balan, France), linear low-density polyethylene (LLDPE), and methacrylic acid grafted polyethylene (MAA-g-LLDPE) as the coupling agent. Aluminum hydroxide—ATH (APYRAL^®^40CD)—was purchased from Nabaltec AG (Schwandorf, Germany), and its median particle size was about 1.5 µm. Three kinds of phosphate low-melting glass powders (LMG1, LMG2, and LMG3) whose composition is shown in Table 1 were synthesized at Corning European Technology Center (Avon, France). The compositions of LMG1, LMG2, and LMG3 correspond to those cited in the Corning patent WO2024081120A1 [34]. The median particle sizes of LMGs are, respectively, 1.9 µm, 2.6 µm, and 2.4 µm. All the materials were used without any pretreatments and were analytically pure.

According to the literature [35,36], the glass transition temperatures of LMG1, LMG2, and LMG3 are supposed to be, respectively, 95 °C, 278 °C, and 167 °C.

### 2.2. Sample Preparation

To remove any moisture, the ATH powder was dried at 80 °C overnight. Low-melting glass powders were used immediately after being taken out of the vacuum-sealed bags. Before incorporation in the polymer matrix, mineral fillers were dry premixed for 15 min using a Stuart SS30 Overhead Stirrer, Dual Torque, 230v from Fisher Scientific (Wien, Austria) equipped with an agitator impellers Anchor Type R1331 from Fisher Scientific (Wien, Austria) with a mixing speed of up to 1500 rpm to achieve a good dispersion of LMG with ATH particles. Afterwards, compounds were made using a Haake PolyLab system with Rheomix 3000 internal mixer from Thermo Fisher Scientific (Illkirch-Graffenstaden, France) which consists of two roller rotors operating at a maximum torque of 300 Nm, enclosed in a mixing chamber with a temperature fixed at 150 °C. The polymer pellets were first introduced in the chamber and mixed for 3 min at 30 rpm. The powder mixture was then added and mixed for 4 min at 30 rpm and finally for 10 min more at 50 rpm. The reference formulation comprised 40% polyethylene (PE)/ethylene–vinyl acetate (EVA) and 60% ATH. In the developing formulations, a part of ATH was substituted by LMG, which gave the following final composition: 40% PE/EVA, 50% ATH, and 10% LMG. To prepare the plates with 3 mm thickness and 100 cm^2^ surface area, a Darragon compression press (Hydro Meca section Texier Dufort Hydraulique, Paris, France) was used where the filled matrix was softened at 150 °C for 2 min then compressed for 3 min with high pressure 150 bars.

### 2.3. Characterization Methods

A cone calorimeter (Fire Testing Technology—FTT, East Grinstead, UK) was used to study flammability in accordance with the ISO 5660 standard [37] (sample dimensions: 100 × 100 × 3 mm^3^). The heat flux was fixed to 50 kW/m^2^ and the ignition was piloted using a spark igniter. The Time To Ignition (TTI), heat release rate (HRR), peak of heat release rate (pHRR), time to pHRR, Total Heat Release (THR) and Maximum Average Heat Rate Emission (MAHRE), mass loss (ML) were measured. Two samples of each formulation were used for testing. A relative error index factor ε was calculated highlighting the good reproducibility of the tests (see Supplementary Materials SM1).

The microstructural analysis of composite materials and their combustion residues following high-temperature exposure was conducted using an SEM Quanta 200F (FEI, Hillsboro, OR, USA) at high magnification. To investigate the thermal behavior of LMG and the LMG/ATH mixture under controlled heating conditions, up to a maximum temperature of 600 °C, the SEM was equipped with a heating stage, enabling in situ monitoring throughout the thermal process.

**Figure 1:**
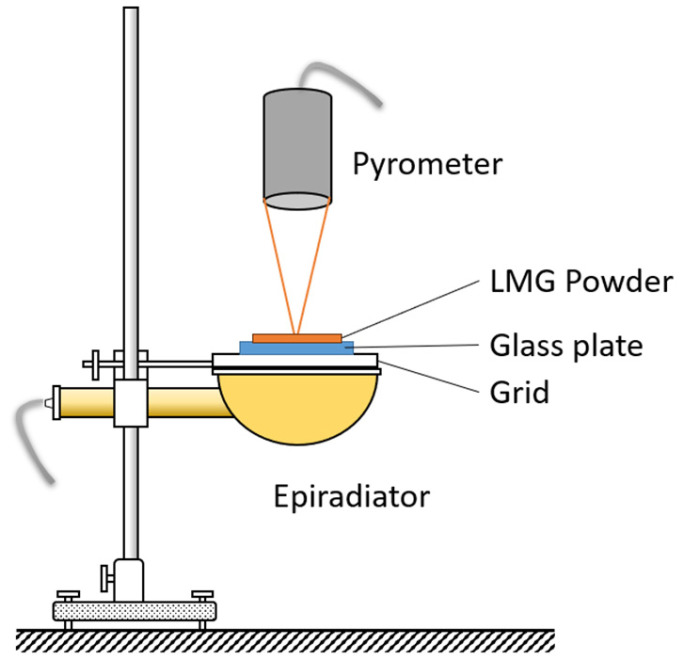
Epiradiator device with temperature measurement.

The structural changes under heating as well as the related change in temperature for LMGs (LMG1, LMG2, and LMG3) and LMGs/ATH mixtures were investigated using an epiradiator as the heating source delivering a heat flux of 30 kW/m^2^ [38]. The experimental setup is shown in Figure 1. A grid was placed above the epiradiator to support a glass slide on which a determined amount of powder (LMG or LMG/ATH mixture) was spread with a uniform thickness. An infrared pyrometer focused on the sample surface enabled the recording of precise temperature measurements.

FTIR spectra of LMG powders were recorded to study the structure of the phosphate network as a function of composition, using the Vertex70 from Bruker (Ettlingen, Germany) in transmission mode at room temperature. A range of 4000–400 cm^−1^ was used to scan samples. All measurements were performed with a 4 cm^−1^ resolution. As a complementary method for the investigation of phosphate network, ^31^P-NMR spectroscopy, was used to collect the chemical shifts in ppm for all LMGs samples and for the mixture of LMG/ATH heated at various temperatures. NMR analyses were performed at the NMR department of the Charles Gerhardt Institute technical platform. The solid-state NMR spectra of ^31^P were recorded on a Varian VNMRS400 spectrometer from Agilent Technologies (Santa Clara, CA, USA) at 400 MHz (9.4 Tesla “Wide Bore” magnet). A Varian T3 MAS (Magic Angle Spinning) probe from Agilent Technologies (Santa Clara, CA, USA) with 3.2 mm ZrO_2_ rotors was used. The measurements were performed with the quantitative Single Pulse technique, with ^1^H decoupling. For these analyses, a 100 s recycle time and a 4 µs π/2 pulse were used. The samples were rotated at a speed of 20 kHz. The chemical shift value was calibrated using potassium dihydrogen phosphate (KH_2_PO_4_) as a secondary reference (4.1 ppm line). The acquisition window is 250 kHz, and the filtering (line broadening) was set between 5 and 50 Hz, depending on the spectra.

A D8 Advance diffractometer from Bruker (Karlsruhe, Germany) with flat-plate geometry and Cu K_α_ radiation was used to undertake X-ray diffraction investigations for the various LMGs and the 50/50 LMG/ATH mixture at different temperatures to analyze the phase crystallization sequence. With a 0.02° scan step and 0.1 s step duration, the acquisitions were captured in the 2θ range between 10° and 60°. To examine how the temperature affects the sample structural evolution, a furnace was positioned around them. At a heating rate of 10 °C/min and a dwell time of 15 min per scan, the analyses were carried every 50 °C for the LMG powders from 25 °C to 600 °C and every 100 °C for the 50/50 mixture from 25 °C to 800 °C. VESTA software version 3.5.8 and Crystallography Open Database Search-Match program were used for phase identification. None of the diffraction patterns matched the entries in the powder diffraction file database, despite the fact that the purpose of the XRD analysis was to identify the chemical makeup of the crystalline phases. Thus, in order to determine these phases, we used VESTA software.

Thermogravimetric analysis (TGA) was used with a Pyris 1 TGA (Perkin Elmer, Shelton, CT, USA) to measure the mass loss of LMG powders, ATH, and that of the 50/50 mixture. The theoretical mass loss was compared to the experimental one to define the possible interaction between both fillers. The analyses were carried out in nitrogen atmosphere, between 30 and 900 °C, at a rate of 10 °C/min.

## 3. Results and Discussion

### 3.1. Flame Retardant Properties of LMG in PE-EVA/ATH

#### 3.1.1. Cone Calorimeter Tests

**Figure 2:**
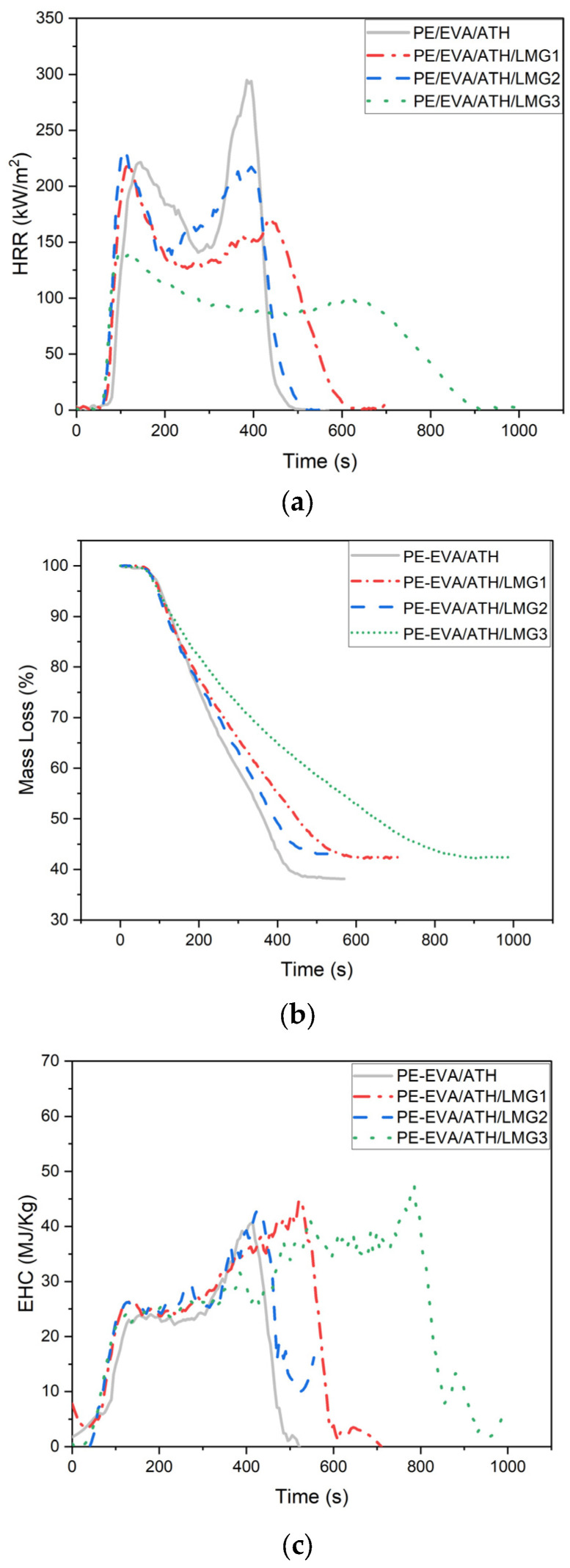
Cone calorimeter data for the various compositions: (**a**) HRR, (**b**) ML, (**c**) EHC.

The Time To Ignition (TTI), first peak heat release rate (pHRR1), second peak heat release rate (pHRR_2_), time to the second peak heat release rate(tpHRR_2_), Maximum Average Heat Release Rate (MAHRE), Total Heat Release Rate (THR), Effective Heat of Combustion (EHC), and the mass loss (ML) of the various PE/EVA-ATH-LMG compositions are presented in Table 2. The HRR, EHC, and ML curves are presented in Figure 2.

The cone calorimeter experiments reveal the different fire behaviors for pure and flame-retarded PE/EVA. As expected, the PE/EVA melted and behaved like a liquid accompanied by numerous bubbles bursting (see Supplementary Materials SM2). At the end of the test, no residue was left, confirming the complete degradation. The HRR curve of the pure polymer shows a continuous growth up from a TTI of around 53 s to a well-defined peak, around 2900 kW/m^2^, beyond which the fall is extremely steep. This gives an indication of a fast combustion process in which, in a very short time, large amounts of heat are liberated, a characteristic of non-flame-retarded material. The sharp peak means that the burning was brisk and all-fast and it corresponds to a thermally thin sample according to the classification of Schartel and Hull [39]. In contrast, the mixture with 40% PE/EVA and 60% ATH develops an entirely different profile during combustion. Firstly, TTI is increased up to around 85 s indicating that ATH delays ignition through water release. Secondly, the HRR curve is flatter and exhibits two peaks. The first peak pHRR_1_ reaches around 220 kW/m^2^ while the second peak (pHRR_2_), occurring at 385 s, was measured at 295 kW/m^2^. This shape is typical of a thick non-charring behavior according to the classification of Schartel and Hull [39]. The second peak is generally assigned to the fact that the decomposition rate increases when the pyrolysis front reaches the rear face, due to adiabatic conditions. This behavior indeed reflects the characteristics of flame retardancy when using ATH: combustion proceeds slower, releasing less heat. The flatter and more extended curve confirm a burning process that is less intense and well-controlled, as is typical for materials bearing improved fire retardancy. This one behaved like a solid without melting. With this sample, a thin and friable mineral layer was left at the end of the test, evidencing the physical and chemical action of ATH [40]. The physical mode of action leads to the formation of a ceramic layer of Al_2_O_3_ that acts as a barrier limiting heat and mass transfer, while the chemical action in the condensed and gaseous phases corresponds to the endothermic dehydration of ATH resulting in dilution of the gaseous fuel. It is noteworthy that the barrier effect is, in this case, very limited.

Let us now examine the curve of blends containing LMGs. The PE-EVA/ATH/LMG1 curve exhibits a slight deviation from the reference, decreasing the pHRR_1_ to 219 kW/m^2^. The second peak (pHRR_2_) appears after a significant delay of 440 s with a reduction to 170 kW/m^2^, indicating that the presence of LMG1 effectively slows down combustion. For this composition, the behavior of the sample turns to thick-charring according to Schartel and Hull [39]. For LMG2, the curve is pretty close to that of the reference, with pHRR_1_ (232 kW/m^2^) being almost similar to that of PE-EVA/ATH and pHRR_2_, occurring at 215 kW/m^2^ after 330 s. In contrast the PE-EVA/ATH/LMG3 curve displays a markedly different profile, characterized by a pHRR_1_ around 142 kW/m^2^ and pHRR_2_ delayed and reduced to 100 kW/m^2^ after 554 s. The shape of the curve is typical of a thick-charring sample as described by Schartel and Hull [39]. The extent of the time interval between pHRR_1_ and pHRR_2_ clearly evidences the rate of the decomposition process and, therefore, gives a clue about the efficiency of the barrier effect, which is beneficial for the fire performance. Hence, the presence of LMG1 or LMG3 improved the efficiency of flame retardants by increasing the time between peaks, likely due to the promotion of a cohesive protective layer that delays fuel release and shields the underlying polymer from high temperatures [33]. Conversely, LMG2 with closer peaks, shows less effective flame retardancy potentially due to a thinner protective layer that allows the second burning phase to follow closely after the first. This variation in behavior highlights the differing efficiency of LMG additives in improving flame retardancy.

Another relevant indicator to assess the barrier effect efficiency is the Maximum Average Heat Release Rate (MAHRE). The MAHRE decreased with the incorporation of LMGs. It dropped from 153.7 kW/m^2^ for the PE/EVA/ATH formulation to 128.2 kW/m^2^ and 90.5 kW/m^2^ for the formulations containing LMG1 and LMG3, respectively. This reduction confirms that LMG1 and LMG3 were more effective in mitigating gas release compared to LMG2 where the MAHRE was 148.6 kW/m^2^.

The mass loss curves (Figure 2b) revealed decomposition kinetics in close correlation with those deduced from HRR curves and discussed before. In terms of final residues, the PE-EVA/ATH composition should theoretically leave 39.2% residue, whereas a composition containing 10% LMG should theoretically leave 42.4% residue. These calculated mass loss values, as shown in Table 2, are consistent with the experimental results. The agreement between theoretical and experimental residue percentages suggests that there is no synergistic effect in terms of char formation; LMG does not act as a char promoter in this system.

The effective heat of combustion (EHC), which corresponds to the THR divided by the mass loss, assesses what happens in the gas phase during the combustion process. As shown in Table 2, with the substitution of 10% ATH by of LMG, EHC is in the same range, increasing slightly by 8–13% compared to ATH in the presence of LMG. The curves of EHC versus time as shown in Figure 2c evidence that a plateau at circa 25 MJ/kg is rapidly reached after ignition. This value is due to the simultaneous release of fuel from PE-EVA decomposition with a high EHC (circa 40 MJ/kg) and of non-combustible water vapor from ATH decomposition. EHC increased at the end of the combustion process up to 40 MJ/kg due to the depletion of water vapor and acetic acid (from EVA) prior to the complete pyrolysis of the matrix. It should be noted that this increase in EHC is relatively steep for ATH and ATH/LMG2-containing compositions, while it is more progressive for ATH/LMG1 and ATH/LMG3 compositions. It means that, in the presence of an efficient protective layer, the pyrolysis rate is controlled even after ATH has released all its water.

The cone calorimeter results highlighted that the partial substitution of ATH by LMG, although not promoting any charring, enhanced the protective effect of the mineral layer, leading to a strong reduction in the heat release rate during the whole burning process. This justifies the reason to investigate with deep attention the combustion residues. The more efficient FR system (ATH/LMG3) was shown to compete advantageously with those found in the literature for cable applications (see Supplementary Materials SM3) [14,15,18,41,42,43,44,45].

#### 3.1.2. Characterization of the Residues

**Figure 3:**
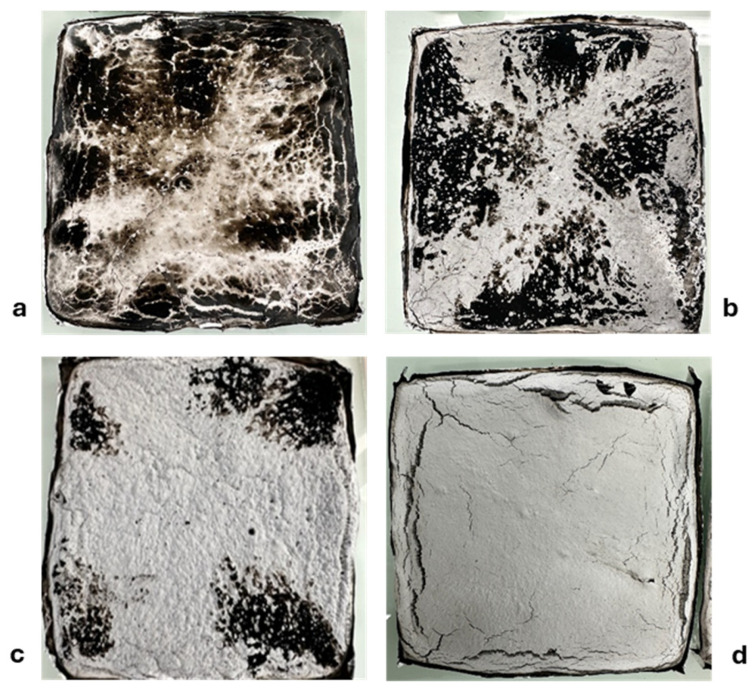
Photos of cone calorimeter residue top surface: (**a**) PE-EVA/ATH, (**b**) PE-EVA/ATH/LMG1, (**c**) PE-EVA/ATH/LMG2, and (**d**) PE-EVA/ATH/LMG3.

**Figure 4:**
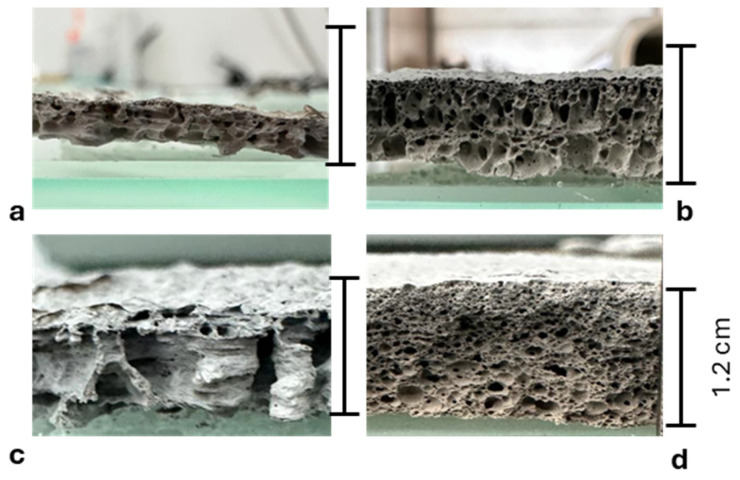
Photos of cone calorimeter residue cross sections: (**a**) PE-EVA/ATH, (**b**) PE-EVA/ATH/LMG1, (**c**) PE-EVA/ATH/LMG2, and (**d**) PE-EVA/ATH/LMG3.

**Figure 5:**
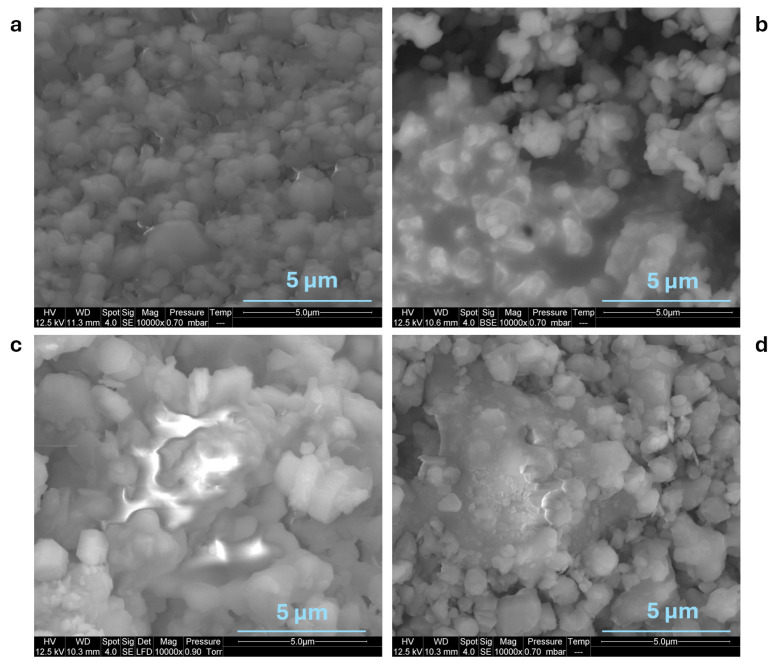
SEM images of cone calorimeter residue top surface (**a**) PE-EVA/ATH, (**b**) PE-EVA/ATH/LMG1, (**c**) PE-EVA/ATH/LMG2, and (**d**) PE-EVA/ATH/LMG3.

**Figure 6:**
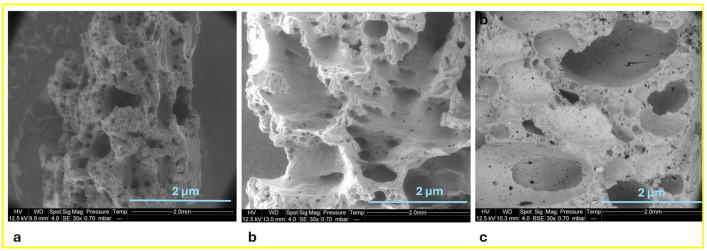
SEM images of cone calorimeter residue cross section (**a**) PE-EVA/ATH/LMG1, (**b**) PE-EVA/ATH/LMG2, and (**c**) PE-EVA/ATH/LMG3.

To understand the improvement in fire behavior in the presence of 10% LMG (LMG1; LMG2; LMG3), the macroscopic and microscopic structures of the residues from the cone calorimeter tests were analyzed by digital images, Figure 3 and Figure 4 and SEM Figure 5 and Figure 6. Photos taken from above the top surface of the residue (Figure 3) show that PE-EVA/ATH with 0.4 cm thickness appear to be dark, non-cohesive and exhibits visible cracks across the surface. It is assumed that the pyrolysis gases are likely to destroy the Al_2_O_3_ layer due to its high brittleness [46]. Introducing LMGs into the formulation reduced cracking. For PE-EVA/ATH/LMG1, the residue surface is mostly white with substantial black charred areas, and the cohesiveness increased, making it easy to handle the residue without it breaking apart. Similarly, PE-EVA/ATH/LMG3 residue was smooth and uniformly white with a few tiny fractures, suggesting a cohesive barrier layer that was well-formed and able to reduce the pyrolysis rate of the underlying material, which is consistent with the obtained HRR curve. PE-EVA/ATH/LMG2, reveals a charred, cohesive, smooth, and uniform layer. However, this formulation was found to be more brittle compared to the two others with LMG1 and LMG3. Note that the absence of black areas on the surface of PE-EVA/ATH/LMG3 may be due to longer burning, promoting the oxidation of char especially when flame starts to vanish.

Photos of the cross sections of residues shown in Figure 4 are evidence that the final mineral layers exhibit a foam-like structure. The pictures enable the assessment of the expansion ratio of the layers after combustion and thus the intumescent effect. The PE-EVA/ATH formulation with a 0.4 cm layer showed a 33% thickness increase compared to its initial state. PE-EVA/ATH/LMG1 (0.9 cm) was found to have expanded by a significant 200%, PE-EVA/ATH/LMG2 (0.7 cm) increased by 133%, while PE-EVA/ATH/LMG3 (1.2 cm) showed a more considerable expansion of 300%. These findings highlight enhanced intumescent behavior with the addition of LMG, particularly in the case of PE-EVA/ATH/LMG3, which demonstrated the most substantial expansion. This underscores the effectiveness of LMG in optimizing fire performance. Additionally, it is clear from the cross section of the residues, as shown in Figure 4, that the type of LMG used in the formulation affects the size and shape of the porosities. Specifically, the PE-EVA/ATH/LMG3 formulation produced a residue with smaller pores compared to PE-EVA/ATH/LMG1. Meanwhile, the PE-EVA/ATH (Figure 5) and PE-EVA/ATH/LMG2 formulations resulted in a residue with larger and heterogeneous pores. According to this, LMG3 and LMG1 in PE-EVA/ATH composites are able to promote a more homogeneous char structure, which may support the integrity of the material during burning [33].

It is important to emphasize that the formulations incorporating LMGs also showed an increased cohesiveness of their residual layer that was absent for the PE/EVA/ATH blend. It is assumed that, during testing, LMGs melt to form a glassy layer, strengthening the barrier. SEM images shown in Figure 5 clearly reveal that LMG particles flowed between ATH particles, gluing the hydrate or alumina particles together. This improved bonding creates a more cohesive and effective barrier against heat and flame, enhancing the overall fire resistance of the formulation.

Figure 6 shows the SEM observation of the cross section of the residue samples. It was very difficult to obtain an observation of a significant thickness for the PE-EVA/ATH residue because of its brittleness, but as for the others and according to the literature, the presence of pores indicates that bubbles formed during burning left these holes. These pores play a role in heat transfer through the matrix. A highly porous structure functions as a thermal insulator by forming a network of air-filled spaces, which are poor heat conductors. This slows down heat transmission and delays the rise in matrix temperature. Furthermore, heat is further hindered from passing through the material by the complicated track that these tiny channels produce. A structure with small holes is beneficial as it better protects against heat transfer, hinders the release of volatiles from the matrix, and delays the degradation of the polymer matrix, resulting in a lower heat release rate [33,47,48].

Overall, even if the use of low-melting glass (LMG) seems beneficial as a flame-retardant synergist, differences in HRR curves, combustion times, MAHRE, residue structures, and barrier layer function show that the nature of the LMG, specifically its chemical composition and behavior, has a major impact on the fire behavior of the various formulations under study. It is essential to investigate the mechanism behind this system when LMG is incorporated into formulations that contain ATH to comprehensively understand how this flame-retardant system affects fire behavior.

### 3.2. Study of the Flame-Retardant Mechanisms

#### 3.2.1. Thermal Behavior of LMG Powders

**Figure 7:**
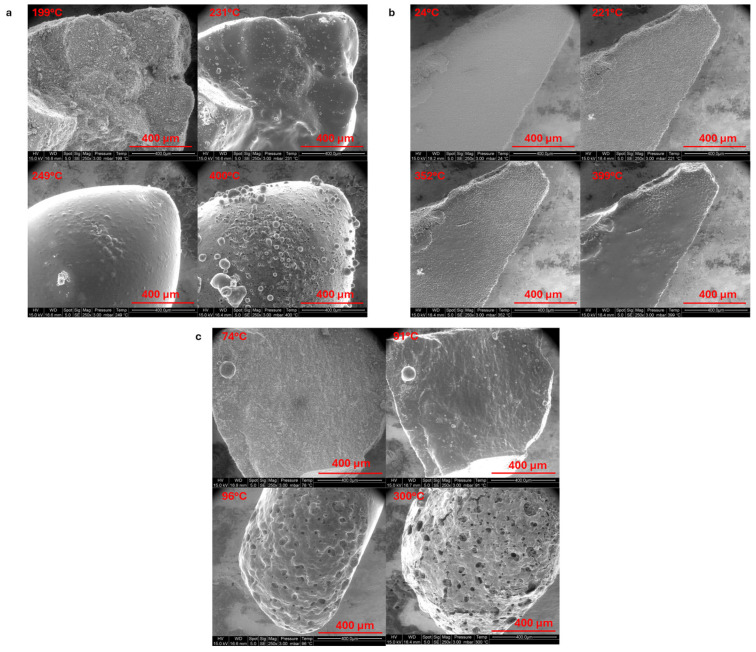
SEM observations of (**a**) LMG1, (**b**) LMG2, and (**c**) LMG3 morphology changes while increasing temperature.

The behavior of LMG powders under heat exposure was examined by gradually increasing the temperature from room temperature to 400 °C, using an integrated heating stage in the SEM chamber (Figure 7). Each LMG has a distinct glass transition temperature (T_g_), and this difference can be identified through several observable phenomena. The powders may become softer, more abrasive, or the particles may begin to agglomerate together as the temperature approaches T_g_. Cracks or changes in how the particles adhere to each other might also appear near T_g_. Beyond 400 °C, sample deformation or motion makes observations difficult. In LMG1, the grains become smoother as the temperature rises, and the granular appearance almost disappears at 250 °C. Significant bubbling and pores creation occur at the highest temperature of 400 °C, suggesting a phase transition or melting process. Even when heated, the granular feature is still observed in LMG2. The surface becomes softer around 400 °C. The LMG3 surface starts to exhibit some evolution and a moderate roughness around 90 °C. As the surface is heated further, it develops many pores and irregularities and becomes noticeably rougher; The phenomenon observed for LMG3 is similar to that of LMG1, but it occurs at a lower temperature. In conclusion, LMG2 displays modest surface modifications, whereas LMG1 and LMG3 show notable morphological changes including flow, bubbling, and pore formation at high temperatures. LMG3 displayed modifications at lower temperatures compared to LMG1 and LMG2. Note that the order of softening (LMG3 < LMG1 < LMG2) is not in accordance with the glass transition claimed in the literature [35,36].

**Figure 8:**
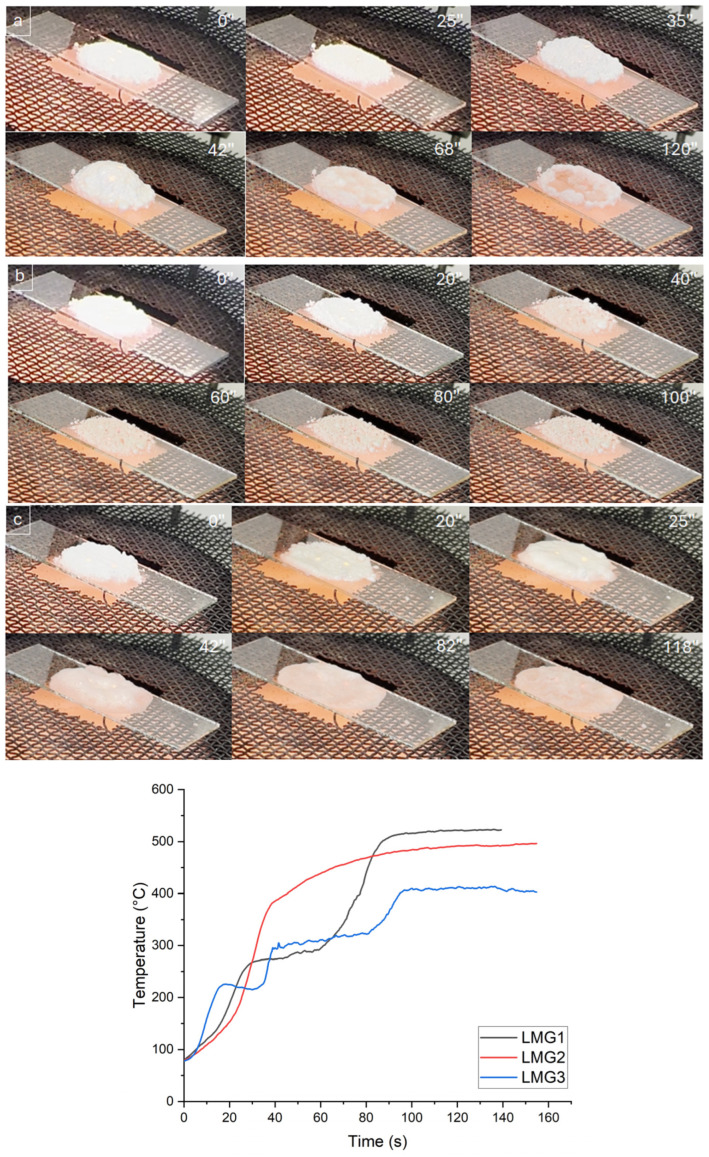
Evolution of the appearance (**top**) and surface temperature (**bottom**) of powders under 30 kW/m^2^ heat flux using epiradiator (**a**) LMG1, (**b**) LMG2, and (**c**) LMG3.

In order to gain a better understanding of what happens when the materials are exposed to heat at a heating rate comparable to that used in the cone calorimeter test, an additional qualitative test was carried out using an epiradiator (Figure 8). It is important to note that the formulations containing LMGs showed a more expanded and cohesive residual layer compared to the PE/EVA/ATH blend, as previously demonstrated. It was assumed that this is due to the melting of LMG particles that bind ATH particles and fortifies the residual layer that acts as shield against heat and gas diffusion. It was hypothesized that the viscosity of the various types of LMG varied with temperature and that it is a key parameter in the ability of LMG to glue ATH particles. The apparent viscosity, or viscous behavior, was evaluated by visual observation of LMG powder beds exposed to a 30 kW/m^2^ heat flux. Videos were acquired and photos were captured at different times during the test. Additionally, the temperature of LMG was measured using a pyrometer placed perpendicular to the glass slide supporting the powder bed. The evolution of LMG temperature is presented in Figure 8.

As for LMG1, the particles started to melt after 25 s, resulting in the formation of a liquid film. According to Figure 8, this phenomenon occurred at a temperature of circa 270 °C which was consistent with what was observed by SEM. Immediately after melting, bubbling was observed, and considering the low viscosity of LMG1, this induced a blowing of the sample. Bubbling and blowing continued up to 65 s and then progressively stopped. During this period, the temperature was almost constant between 270 and 290 °C. After the bubbling stopped, the sample deflated, and the temperature increased again up to a plateau at 520 °C. The sample mass was measured before and after the test and a mass loss of 24.7 wt% was determined. As far as LMG2 is concerned, the particles started melting after circa 40 s corresponding to a small plateau at a temperature of around 385 °C. Once again, this value is close to that measured during SEM observations. Contrary to LMG1, in the molten state, LMG2 particles did not form a film but rather aggregated to form big clusters. Once these clusters were formed, the structure of the sample did not evolve anymore, and the temperature reached a plateau at around 500 °C. In the case of LMG2, the mass loss during the test was measured to be 10.4 wt%. Concerning LMG3, the particles started melting after 18 s, reaching a plateau at a temperature of 225 °C. Consistent with SEM observations, this was the lowest melting point among all LMGs. Once melted, the particles form a low-viscosity liquid film that spread on the glass slide. After a few seconds, the liquid film started bubbling and the temperature increased up to a plateau at circa 300 °C. A vigorous bubbling was observed and the sample inflated. After a period of 40 s, the bubbling stopped, and the temperature increased again up to a final temperature of around 410 °C. For LMG3, a 14.0 wt% mass loss was evidenced during the test.

From those tests, several conclusions can be drawn. Firstly, the melting temperature was found to increase in the order of LMG3 < LMG1 < LMG2. Secondly, the apparent viscosity, assessed as the ability of the liquid to spread over the slide, also increases in the order of LMG3 < LMG1 < LMG2. It could be observed that the lower the viscosity and the melting temperature, the higher the heating rate in the first moments of the test. It may be assumed that those behaviors are closely related to the LMG structure, i.e., the ionic radius, ionic charge or coordination environment and the interaction that could happen within the network. Compared to monovalent cations like sodium (Na^+^) and potassium (K^+^), divalent cations like zinc (Zn^2+^) interact with the phosphate groups in a different way. Divalent cations generate higher interactions (higher field strength) due to their higher charge and, consequently, the melting temperature as well as the viscosity in the molten state are enhanced. Regarding monovalent cations, since potassium (K^+^) has a bigger ionic radius than sodium (Na^+^), there may be less contact within the network and, consequently, lower melting temperature and viscosity. The case where LMG3 with K^+^ had the lowest viscosity of all the cations examined supports this phenomenon. Compared to Na^+^ and Zn^2+^, the capacity of K^+^ to interact is limited by its greater ionic size and monovalent charge. So, although cation molar mass plays a part in determining the total viscosity of the solution, ionic charge and size also have a significant impact on how viscosity behaves in cation-containing solutions [49].

The above-described experiments highlight the temperature range of glass transition, during which the glass softens gradually [50]. Actually, the glass transition temperature of phosphate glasses primarily depends on the cross-link density, average length of the phosphate chain, and the bonding strength of the structure. In other words, the observed behavior connects directly with the phosphate network structure and composition [51]. Several authors have thoroughly investigated the impact of various cations on the structural alterations in phosphate glasses and melts [49,52,53]. To determine the cause of the behavioral differences that may provide a clear explanation for the greater effectiveness of the barrier layer generated during the fire test, the structural characterization of the three types of LMG was carried out.

#### 3.2.2. Structural Characterization of LMG Powders

##### NMR Spectroscopy

**Figure 9:**
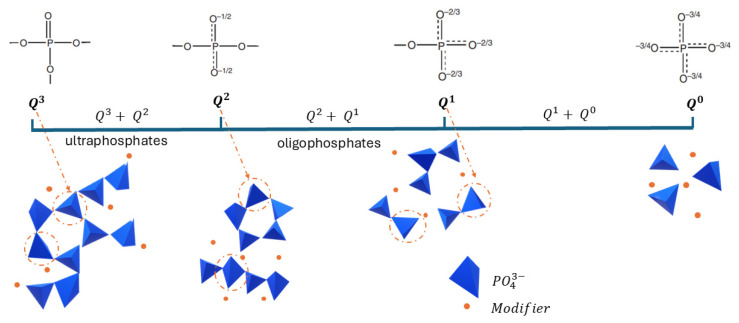
Phosphate basic building brick.

**Figure 10:**
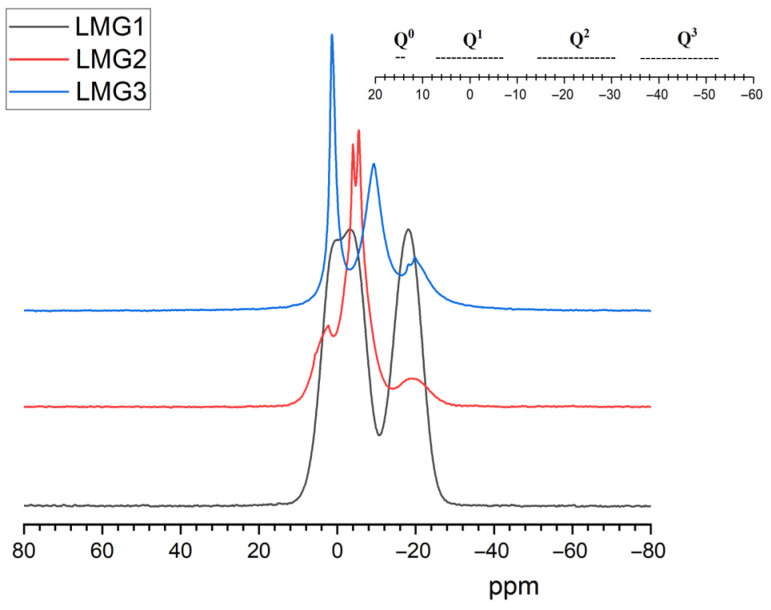
^31^P-NMR spectra of LMG1, LMG2 and LMG3.

^31^P-NMR is a technique well adapted to study the structure of phosphate glasses. According to Parsons [54], the structure of phosphates consists of corner-to-corner connected tetrahedral PO_4_ groups forming chains and networks. This can be described from four different possible configurations of PO_4_ tetrahedron which are known as Q^0^, Q^1^, Q^2^, and Q^3^ depending on the number of bridging oxygen atoms (Figure 9). The ^31^P-NMR spectra in Figure 10 shows that phosphate glasses exhibit a highly sensitive chemical shift (CS) of the ^31^P nuclei depending on the kind of modifier cations bound to the non-bridging oxygens. LMG1 showed a doublet peak in the CS range [2; −5 ppm] with a maximum at 0.4 ppm and −3.4 ppm and one broad peak around −17 ppm, which can be attributed to the Q^1^ and Q^2^ structural units, respectively. The small splitting observed for the first peak could be caused by the existence of non-equivalent sites of Q^1^, with different neighboring related to the presence of three non-bridging oxygen atoms [50,55,56,57]. For LMG2, a potassium–sodium–zinc phosphate glass, signals are observed at CS of 2.5 ppm, −4 ppm, −5.4 ppm, and −20 ppm. The peak with the highest intensity corresponds to the doublet observed at −4 ppm and −5.4 ppm, that can be attributed to Q^1^ units. The peak at 2.5 ppm with a medium intensity can be attributed to Q^0^ units while the smallest peak at −20 ppm is related to Q^2^ units. As for LMG3, it exhibits four distinct resonances at CS of 1.2 ppm, −9.5 ppm, −18.3 ppm, and −19.9 ppm. The main peak occurs at 1.2 ppm and is characteristic of the presence of Q^0^ units. This is followed by a peak at −9.5 ppm related to Q^1^ units and finally a doublet at −18.3 and −19.9 ppm corresponding to the two non-equivalent Q^2^ sites.

From the three spectra, it can be concluded that LMGs contain relatively short phosphate chains since Q^1^, which corresponds either to pyrophosphate or to the polyphosphate chain-end, is the major unit. Conversely, Q^2^ which corresponds to the unit inside the polyphosphate chains exhibits a small intensity resonance. These results are consistent with the Van Wazer theory that states that the addition of alkali oxide depolymerizes the three-dimensional P_2_O_5_ network [57]. Moreover, this effect is all the greater as the radius of the cation is large [58].

##### FTIR Spectroscopy

**Figure 11:**
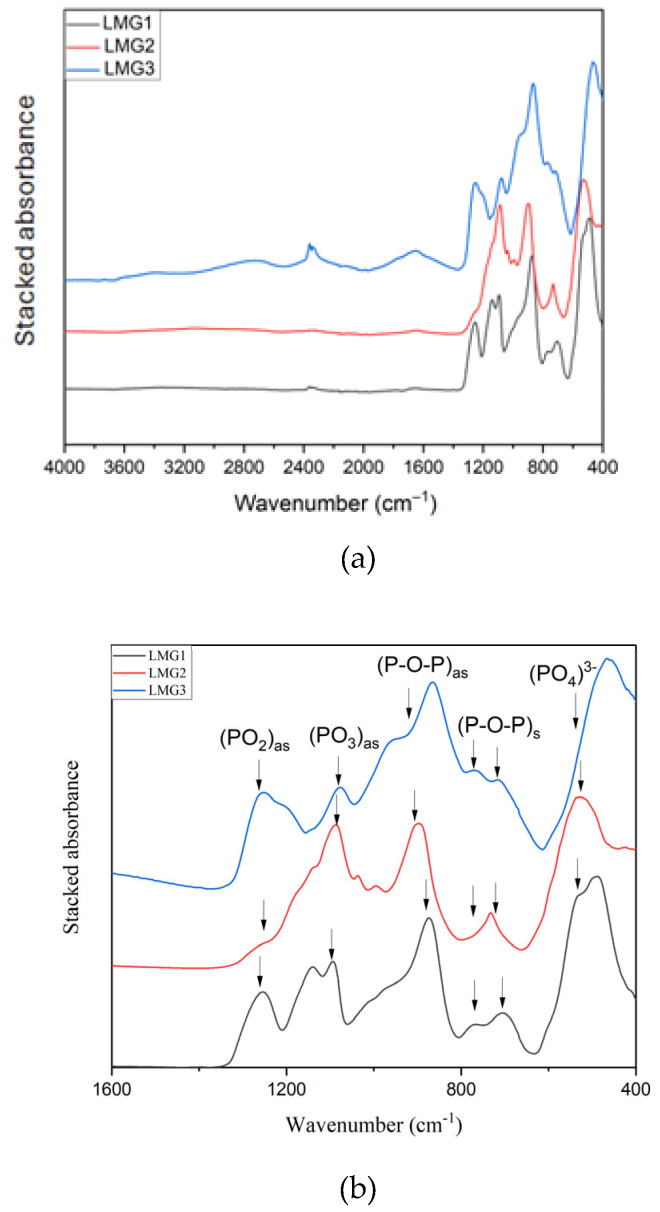
(**a**) FTIR spectra of LMG1, LMG2, and LMG3; (**b**) zoom in the 1600–400 cm^−1^ range.

The FT-IR spectra of the three LMG powders are shown in Figure 11. It can be observed that the LMG samples exhibit relatively similar IR absorption spectra, likely because their composition is based on phosphate and spectra expresses the main features of phosphorus–oxygen networks. Two groups of broad bands in the range 1660–1620 cm^−1^ and 3700–3200 cm^−1^ are indicative of either the presence of free water in these LMG or O-H bonds in phosphate units.

Figure 11 shows a zoom within the 1600–400 cm^−1^ range that corresponds to the phosphate lattice vibrations. The bands between 400 and 500 cm^−1^ can be linked to the bending vibrations of bridging phosphorus, like O-P-O. The symmetric stretching of P-O-P bridges is responsible for the absorption bands at 776 and 714 cm^−1^ [59]. The asymmetric stretching of P-O-P bridges is responsible for the band observed at 900 cm^−1^ [32,60,61,62,63]. The asymmetric stretching of PO_3_^2−^ groups, which is characteristic of Q^1^ structural units (chain-end groups), is responsible for the absorption band observed around 1095–1063 cm^−1^ [59,64]. The band observed at 1260 cm^−1^ can be attributed to the asymmetric stretching vibration band of the non-bridging oxygen atoms bonded to phosphorus atoms (PO_2_) and also to the asymmetric stretching of the P=O bond [65].

It should be underlined that the LMG2 FTIR spectrum, even though it shows the main features of phosphate glasses, exhibits some significant differences with LMG1 and LMG3. In LMG1 and LMG3, the peak around 1260 cm^−1^ is highly noticeable, signifying the substantial existence of the asymmetric stretching vibration of O-P-O and P=O [59,64]. In LMG2, the inclusion of ZnO causes a decrease in the intensity of this band or a shift towards lower wavelength. This is likely due to the intense electric field of zinc ions with higher charge and smaller radius compared to potassium or sodium ions.

##### X-Ray Diffraction

**Figure 12:**
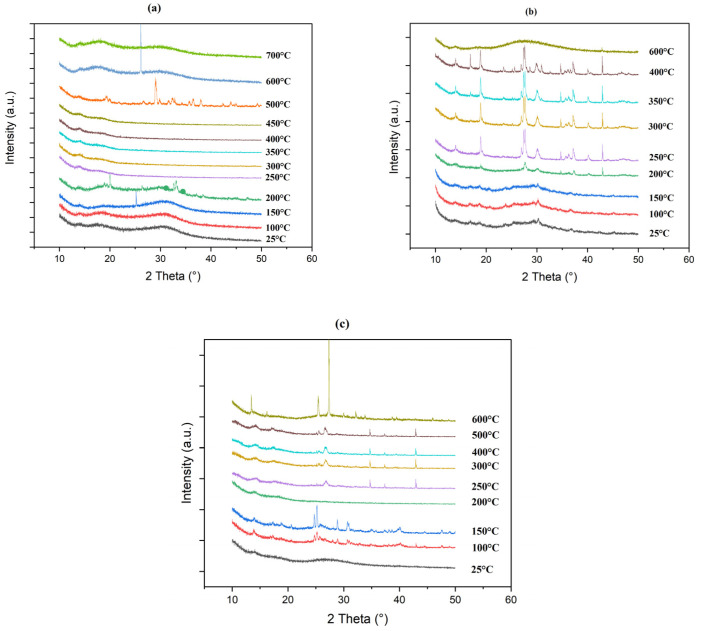
XRD patterns of (**a**) LMG1, (**b**) LMG2, and (**c**) LMG3 at different temperatures.

X-Ray diffraction (XRD) study was carried out to investigate the structural changes in glass powders exposed to heat. This research was conducted in addition to the knowledge obtained through epiradiator testing and scanning electron microscopy (SEM) in conjunction with a furnace. The behavior of the phosphate network under heat could be fully investigated thanks to this technique. The organization and evolution of the phosphate species were determined by comparing these results with spectroscopic data from FT-IR and NMR, providing a thorough insight of the changes taking place within the glass matrix. Figure 12 shows the evolution of XRD pattern for LMG1, LMG2, and LMG3 when temperature increased from 25 to 600 °C. For the three types of LMG, noticeable changes were observed. For LMG1, the presence of two widespread bumps indicated that the initial amorphous structure was kept from 25 °C up to 200 °C. The peak detected at 150 °C was attributed to a disturbance linked to the support substance. While the majority of the material remains amorphous, slight changes in the diffraction patterns suggest that some small crystals could have been formed as the temperature increased from 200 °C to 500 °C. In contrast, above 500 °C, the material reverts back to an amorphous state which corresponds to the molten state. For LMG2, between 25 °C and 150 °C, the diffraction patterns show wide and indistinct peaks, suggesting that the material remained in a glassy state. Once the temperature reaches 200 °C, sharper peaks appear in the XRD pattern together with the broad amorphous background, indicating the onset of crystalline phase formation. At elevated temperatures ranging from 350 °C to 600 °C, this phenomenon becomes more noticeable as clear and strong peaks start to form, highlighting substantial crystallization occurring in LMG2 structure. At 600 °C, LMG2 reaches its molten state, and the structure turns again to amorphous. As for LMG3, the initial patterns exhibit large bumps at lower temperatures typical of the amorphous nature of the material. From 100 to 150 °C, some sharp peaks witness the formation of small crystals that completely disappeared at 200 °C. From 250 °C to 600 °C, the XRD profiles show some sharp peaks, different from those observed at a lower temperature, which indicated that crystalline phase was formed. Contrary to LMG1 and LMG2, it was remarkable that LMG3 was not in the molten state at 600 °C.

**Figure 13:**
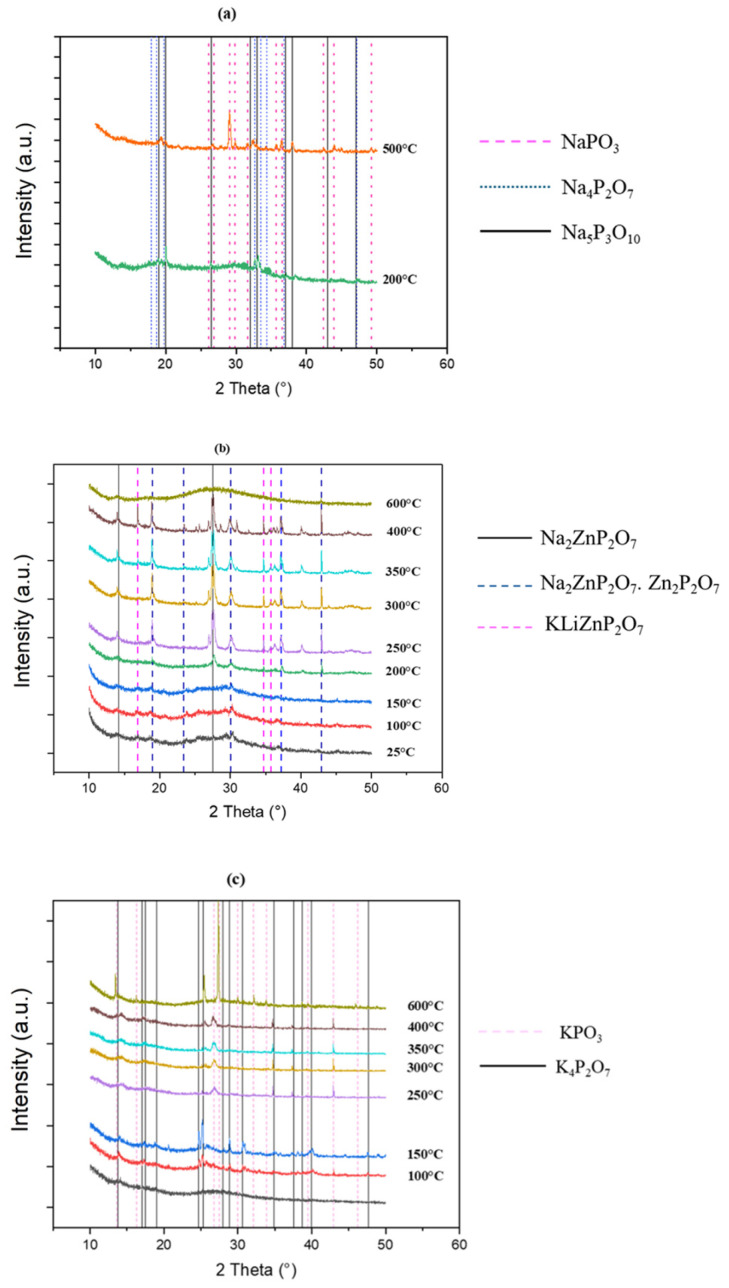
Crystalline phases identification in XRD patterns of (**a**) LMG1, (**b**) LMG2, and (**c**) LMG3.

The primary crystallization products for LMG1 at 200 °C, as shown in Figure 13, are Na_4_P_2_O_7_ and Na_5_P_3_O_10_. At 500 °C, a third crystalline species NaPO_3_ emerges, coexisting with the previously identified phases. The Q units undergo significant changes as it shifts from sodium pyrophosphate (Na_4_P_2_O_7_) and sodium triphosphate Form II (Na_5_P_3_O_10_), [66] to sodium metaphosphate (NaPO_3_). Q^1^ units are contained in Na_4_P_2_O_7_, with each phosphorus atom being linked to two bridging oxygens and two terminal oxygens, showing the presence of pyrophosphate structures. Na_5_P_3_O_10_ has a more intricate structure including Q^2^ units, in which phosphorus atoms are connected to two bridging and two non-bridging oxygens, a feature of triphosphate chains. In NaPO_3_, the arrangement consists of Q^2^ groups, characteristic of metaphosphates. According to Banach et al. [66], at a temperature above 450 °C, the sodium triphosphate transforms from Form II to Form I.

For LMG3, at 100 °C and 150 °C, K_4_P_2_O_7_, which is pyrophosphate, predominantly appears, accompanied by a small amount of KPO_3_ metaphosphate where this phase begins to predominate starting at 250 °C [67].

For LMG2, peaks that characterize the crystalline phases are correspond to Na_2_Zn(P_2_O_7_) phase, Na_2_ZnP_2_O_7_.Zn_2_P_2_O_7_, and KLiZnP_2_O_7_ [68], all along with the presence of certain species also identified with LMG1 and LMG3. Lapshin et al. [69] noted that at temperatures around 250 °C to 300 °C, crystalline peaks correspond to α-LiKZnP_2_O_7_ (monoclinic) and β-LiKZnP_2_O_7_ (rhombic). Due to their structural similarity, the transition between these forms occurs rapidly and is difficult to distinguish [70]. Around 500 °C we may have an interaction that happens between compounds Na_2_ZnP_2_O_7_ and K_2_ZnP_2_O_7_ leading to another crystalline species, NaKZnP_2_O_7_ [69].

### 3.3. Study of LMG-ATH Interactions

Section 3.1 has revealed the essential role of LMGs in enhancing the flame retardancy of PE-EVA/ATH compositions. The fire performance was enhanced by the creation of a more cohesive protective layer, which slowed down the polymer decomposition and thus the power of the fire. This effect is likely due to the interactions occurring between LMG and ATH. Therefore, it is relevant to study in more detail these interactions in a high-temperature environment.

#### 3.3.1. SEM Observations

**Figure 14:**
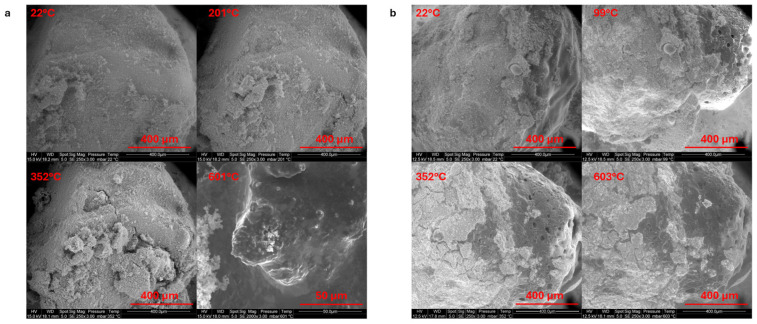
SEM observations of (**a**) ATH-LMG1 and (**b**) ATH-LMG3 mixtures while increasing temperature.

A similar procedure to the one presented in Section 3.2.1 was used to examine the evolution of LMG/ATH (50/50) powder mixture under heat exposure using SEM. Figure 14a (LMG1/ATH) shows a granular, rough surface that holds onto its texture until 200 °C, suggesting a delayed transition to a more homogenous phase in comparison to LMG1. Indeed, LMG1 shows almost undetectable granularity at 250 °C (Figure 7). Partial melting of LMG1 is shown at 500 °C, resulting in smoother textures as LMG1 starts to move and circulate amongst ATH particles. LMG1 completely envelops the ATH particles as the temperature rises to 600 °C, indicating that ATH has integrated into the LMG1. The thermal behavior of LMG3 in combination with ATH is not the same as that of LMG3 alone when temperatures are raised (Figure 14b). The combined material seems reasonably smooth at first, similar to LMG3 alone, at lower temperatures. However, the surface of LMG3 alone begins to exhibit minor variations and moderate roughness as the temperature rises, especially beyond 90 °C. As the heat increases, pores form, and the surface becomes even rougher. The blend of ATH and LMG3, on the other hand, exhibits a more regulated surface morphology with reduced roughness and porosity, suggesting that ATH aids in stabilizing the surface. ATH particles are observed to be embedded into LMG3.

#### 3.3.2. Thermogravimetric Analysis

**Figure 15:**
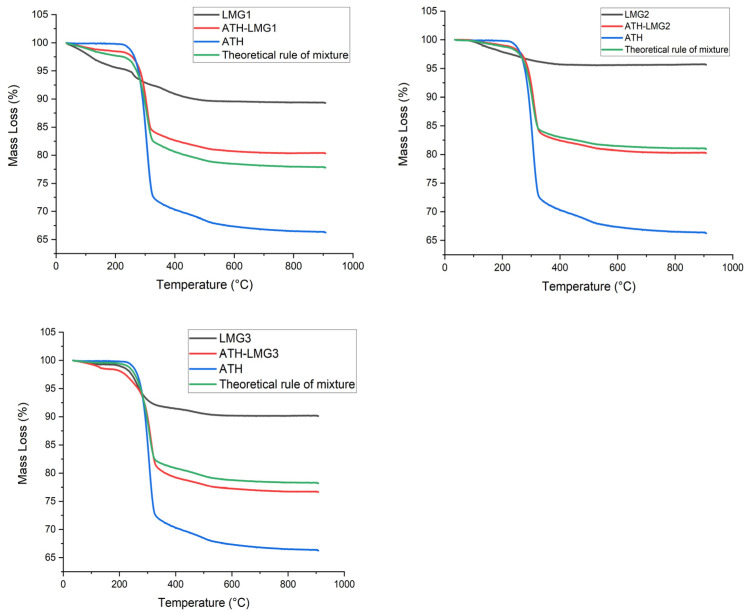
TGA curves of ATH, pure LMGs, and ATH-LMG mixtures.

To evaluate the thermal decomposition of powders, thermogravimetric analysis (TGA) was performed. Figure 15 shows the mass loss curves of ATH, pure LMGs, as well as a 50/50 ATH/LMG mixture. According to the findings, ATH starts to decompose at a temperature of about 200 °C. The ATH decomposition occurs in two steps: a main step between 200 °C and 300 °C which corresponds to the transition from trihydrate to monohydrate with the loss of two water molecules (Equation (1)) and a second step between 300 °C and 550 °C that corresponds to the dehydration of the monohydrate (Equation (2)) as previously described by Zhu [71].

Al_2_O_3_.3H_2_O → Al_2_O_3_.H_2_O + 2H_2_O (1)

Al_2_O_3_.H_2_O → Al_2_O_3_ + H_2_O (2)

As for LMG1, the experiment revealed that heating resulted in three small mass loss events with a total mass loss of 11% at 900 °C. The first mass loss occurs in the range 45–152 °C. This step can be attributed to the release of water adsorbed on LMG surface. The second and third steps occur, respectively, in the range 240–280 °C and 350–435 °C. It is likely that these steps correspond to the partial decomposition of the glass itself. For LMG2, there are two mass loss events: the first occurs between 75 and 205 °C and is rather small, while the second is broader and occurs between 240 and 350 °C, with a total mass loss of about 4.3%. Concerning LMG3, three mass loss steps in the ranges 50–150 °C, 230–300 °C, and 425–505 °C with an overall mass loss of around 9.8%. Similar to LMG1, the first step could be attributed to dehydration while the two others correspond to the partial glass decomposition. It should be noted that the hierarchy in total mass loss is similar to that observed during the epiradiator test (LMG1 > LMG3 > LMG2); however, the magnitude of mass losses is different, indicating that the heating rate may play a role in LMG decomposition kinetics.

Three separate mass loss events were seen in the ATH-LMG1 combination: a narrow loss between 44 and 146 °C, a wide loss between 278 and 326 °C, and a barely perceptible loss between 460 and 530 °C with a total mass loss of 20%, meanwhile it was 34% for ATH. Likewise, three stages of mass loss were seen by ATH-LMG2: a narrow loss between 84 and 158 °C, a wide loss between 280 and 333 °C, and a hardly noticeable loss between 463 and 541 °C with a total mass loss around 20%. Three mass loss events were also noted for ATH-LMG3: a little loss between 52 and 147 °C, a wide loss between 283 and 331 °C, and a barely observable loss between 460 and 524 °C with a total mass loss of 23%

The theoretical mass loss of blends was calculated assuming a rule of mixture (no interaction between components). The mismatch between the experimental and theoretical curves is likely to highlight the interaction between LMGs and ATH during decomposition.

Mass Loss theoretical (%) = 0.5 ∗ Mass Loss (ATH) + 0.5 ∗ Mass Loss (LMG)

For the ATH-LMG1 blend, the experimental mass loss is 2.5% lower than the theoretical one. The main mismatch occurs during the second step of the decomposition of LMG1. Therefore, interaction between ATH and LMG1 may reduce the decomposition of the phosphate structure slightly. For the ATH-LMG2 blend, there is almost a perfect agreement between experimental and theoretical curves, indicating no interaction. Finally for the ATH-LMG3 blend, the experimental mass loss is slightly higher than the theoretical one (2%). The main mismatch occurs in the first step where LMG3 loses water; therefore, the mass loss difference could be related to a difference in the initial water content of the samples. On the whole, no great changes in decomposition kinetics can be highlighted when combining ATH and LMGs.

#### 3.3.3. X-Ray Diffraction Analyses

At room temperature, ATH exhibits a monoclinic crystal structure typical of the gibbsite mineral form. From 200 °C, gibbsite starts releasing water and is transformed into boehmite with an orthorhombic crystal structure. At high temperature 800 °C, ATH has entirely turned into aluminum oxide Al_2_O_3_ (*γ*-alumina) with a cubic crystal structure. However, it should be noticed that *γ*-alumina is poorly crystallized as proven by the large width of diffraction peaks [72].

**Figure 16:**
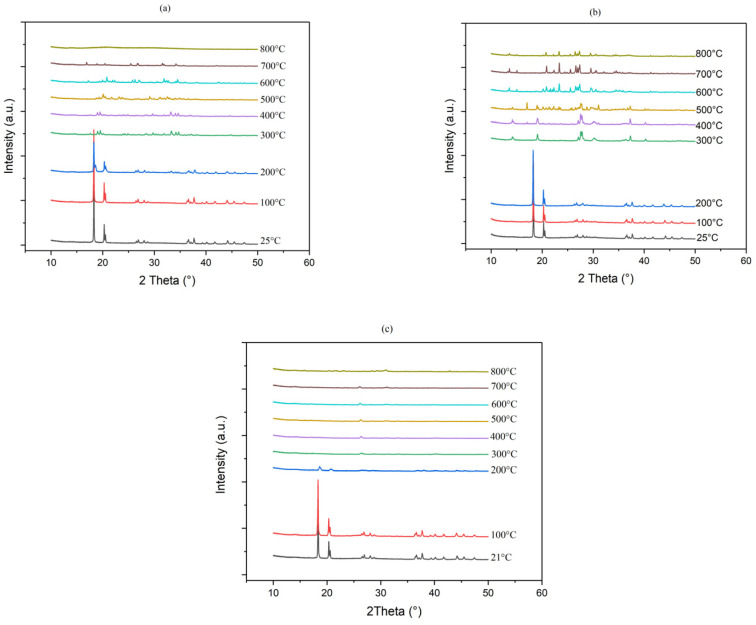
XRD patterns for (**a**) ATH-LMG1, (**b**) ATH-LMG2, and (**c**) ATH-LMG3 at different temperatures.

**Figure 17:**
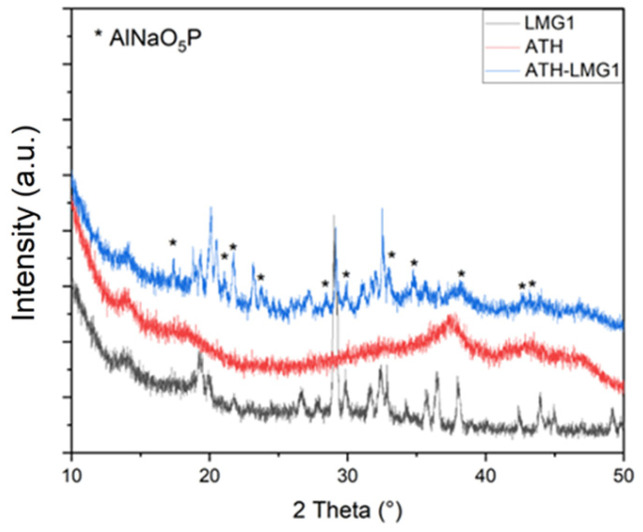
Identification phases in XRD pattern at 500 °C for ATH-LMG1.

Figure 16a showed that the combination of ATH and LMG1 caused a delay in the crystallization process of LMG1. The two crystalline species, Na_5_P_3_O_10_ and Na_4_P_2_O_7_, observed from 200 °C in pure LMG1 (Figure 12) were not seen at this temperature in the mixture. Only boehmite was noted. At 500 °C, the three crystalline species Na_5_P_3_O_10_, Na_4_P_2_O_7_, and NaPO_3_ were present in addition to boehmite (Figure 17). Furthermore, additional crystalline peaks were found and attributed to AlNaO_5_P, suggesting an interaction between ATH and LMG1. The explanation for this is that when the temperature rises, the ATH undergoes a partial metamorphosis from gibbsite to boehmite to *γ*-alumina. During this transition, certain surface-OH groups are replaced by phosphorous groups, forming P-O-Al bonds that cause the crystallization process of LMG1 to be delayed [73].

**Figure 18:**
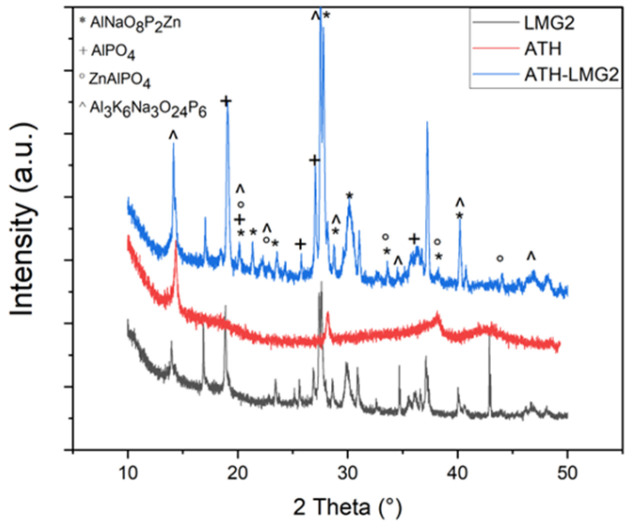
Identification of phases in XRD pattern at 400 °C for ATH-LMG2.

When LMG2 is coupled with ATH, its crystallization does occur at the expected temperature, i.e., from 250 °C (Figure 16b). However, from 500 °C a few more peaks appear especially in the 2theta ranges between 20–25° and 30–35°. These are most likely the outcome of boehmite and phosphate species interactions. When boehmite dehydrates into alumina, it is able to enter the phosphate network as AlO_4_ or AlO_6_. The modifier oxides, Na_2_O and K_2_O, depolymerize the phosphate network, while alumina create Al-O-P bonds, thus stabilizing the network and eventually leading to new crystalline phase formation such as Al_3_K_6_Na_3_O_24_P_6_, AlNaP_2_O_8_Zn; AlPO_4_, and ZnAlPO_4_ in small amounts as highlighted by the diffraction pattern in Figure 18 [74,75].

For the combination between ATH and LMG3, the same process is occurring here as mentioned before. At 300 °C, Figure 16c, weak peaks appear at 29° and 34°, attributed to β-KAlO_2_, which can crystallize when boehmite releases water [76]. Additionally, the presence of KAl(HPO_4_)_2_·H_2_O was also identified in the XRD pattern. However, notable alterations were noted as the temperature rose, especially around 600 °C [77], along with the potential appearance of KPO_3_. At approximately 600 °C as shown in Figure 16c, a notable phase transition occurred. The XRD patterns indicated that KAl(HPO_4_)_2_·H_2_O decomposed, leading to the formation of K_3_PO_4_ (potassium phosphate) and Al_2_O_3_ [78]. It was assumed that the viscosity of the glass, which is known to restrict the structural rearrangements that lead to crystallization and result in the formation of unknown and/or metastable crystalline compounds, affected and controlled the crystallization mechanism that occurred at 400 °C [56].

#### 3.3.4. ^31^P NMR Spectroscopy

**Figure 19:**
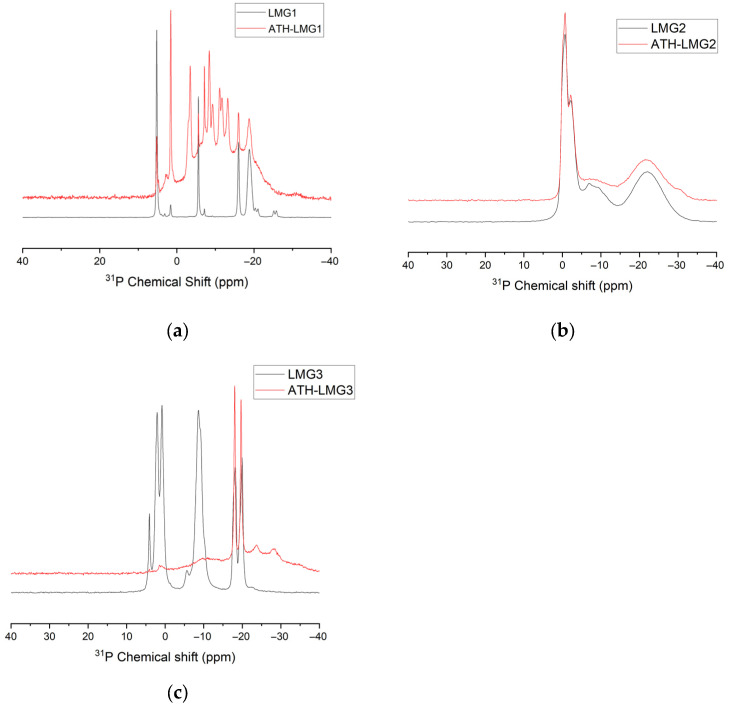
^31^P NMR spectra of LMG and ATH-LMG after heating at 500 °C, (**a**) LMG1, (**b**) LMG2, and (**c**) LMG3.

Figure 19 showed the ^31^P MAS NMR spectra of LMG1 and ATH-LMG1 blend after they were heated to 500 °C at 10 °C/min and then cooled to room temperature. For LMG1, several defined narrow signals appear between 5 and −25 ppm with different intensities indicating the presence of crystalline phases. This result is consistent with XRD results where three different crystalline phases were observed. The sharp signals with the highest intensity at the chemical shift between 5 and −10 ppm can be attributed to Q^0^ and Q^1^ sites, respectively, surrounded by sodium ions; Q^1^ defines Na_4_P_2_O_7_ and the two terminal phosphorus atoms of Na_5_P_3_O_10_ and those around −20 ppm are characteristic of Q^2^ sites attributed to NaPO_3_ and the middle phosphorus atom in Na_5_P_3_O_10_ [79]. The presence of the Q^0^ unit, i.e., orthophosphate PO_4_^3−^, surrounded by sodium ions indicates the presence of Na_3_PO_4_ crystalline species at this temperature.

The ^31^P-NMR spectrum for the ATH-LMG1 mix revealed the appearance of new peaks corresponding to new crystalline species, which confirm the idea that an interaction happens between these two components at high temperature. The effect of Al_2_O_3_ on phosphate glass is the formation of P-O-Al bonds, strengthening the structural network leading to the so-called sodium aluminophosphate [80]. The chemical shifts observed for ATH-LMG1 after heating at 500 °C are consistent with the presence of aluminophosphate or xAl_2_O_3(1−x)_ NaPO_3_. The new peaks, which emerge at −8.5 and −13.1 ppm, are associated with Q^1^ aluminophosphate groups, which arise when P-O-Al connections take the place of P-O-P linkages.

For LMG2, the spectrum is characterized by broader signals with two main resonance peaks around 0 and −20 ppm, suggesting the coexistence of Q^1^ and Q^2^ phosphorus environments, most likely attributed to sodium polyphosphate species. Compared to LMG1, the spectrum of LMG2 displays lower crystallinity, indicating a higher proportion of amorphous phosphate phases. Upon blending with ATH, the ATH-LMG2 spectrum shows a small decrease in the intensity of the Q^2^ site resonance. These changes suggest partial interaction between ATH and the phosphate network of LMG2, leading to the formation of some aluminophosphate species. However, consistently with XRD spectra, the extent of this transformation remains limited, as evidenced by the preservation of the original phosphorus environments.

In contrast, the spectrum of LMG3 presents several sharp and well-defined peaks between 0 and −25 ppm, indicative of highly crystalline phases, in agreement with XRD data. After the addition of ATH, significant spectral changes are observed: the initial sharp signals largely disappear, replaced by broader peaks, characteristic of a more disordered phosphorus network. This suggests a strong interaction between ATH and the phosphate components of LMG3.

Overall, the comparison of LMG1, LMG2, and LMG3 systems highlights different degrees of interaction with ATH at high temperatures. While LMG1 and LMG3 exhibit substantial structural transformations, LMG2 shows only partial reorganization, likely due to its initially more amorphous nature and possibly lower reactivity toward Al_2_O_3_. The interactions between LMG and ATH are likely to participate in the cohesion of the residual layer formed during burning and thus to its flame-retardant efficiency.

## 4. Conclusions

The incorporation of phosphate-based low-melting glass as a synergist in PE/EVA/ATH enhanced the flame-retardant performance of the compositions. The cone calorimeter results showed that LMG3 (potassium phosphate LMG) led, by far, to the best performance with reduced pHRR and MAHRE. The efficiency of the flame-retardant system can be attributed to interactions between LMG and ATH during burning, leading to the formation of an expanded and cohesive layer that protects the underlying polymer and slows down the degradation rate. LMGs interact physically first by melting and diffusing within the ATH particles, leading to the gluing of the hydrated fillers before or during their dehydration. The ability of LMG to flow and glue ATH particles was evidenced to be related to their glass transition temperature and viscosity which are properties closely dependent on the glass network structures. NMR revealed that phosphate LMGs possess short phosphate chains due to the depolymerizing effect of alkaline cations. LMG3 was highlighted to exhibit the lowest Tg and viscosity due to the lower ionic field strength and higher ionic radius of K^+^ compared to Na^+^ and Zn^2+^. LMGs also interact chemically with ATH as proven by the appearance of new crystalline species in XRD patterns. Whatever the LMG, crystalline phases containing P and Al were evidenced. Crystallization may also play a role in the cohesion and efficiency of the protecting layer during burning.

## Figures and Tables

**Table 1:** LMG chemical composition.

Oxide	LMG1 (%mol)	LMG2 (%mol)	LMG3 (%mol)
P_2_O_5_	65	40	65
Na_2_O	35	12	
K_2_O		7.5	35
ZnO		29.9	
Li_2_O		10.6	

**Table 2:** Results of cone calorimeter tests.

Formulation	TTI (s)	pHRR1 (kW/m^2^)	pHRR2 (kW/m^2^)	tpHRR2 (s)	MAHRE (kW/m^2^)	THR (kJ/g)	EHC (kJ/g)	ML_exp_ (%)	ML_calc_ (%)
PE-EVA	53	2891	-	-	673	43.4	43.4	0	0
PE-EVA/ATH	83	220	295	385	154	14.9	24.1	38.1	39.2
PE-EVA/ATH/LMG1	75	219	170	440	128	15.6	27.3	42.2	42.7 *
PE-EVA/ATH/LMG2	60	232	215	370	149	14.9	26.1	42.9	42.7 *
PE-EVA/ATH/LMG3	66	142	100	620	90	15.9	27.4	42.4	42.7 *

* ML_calc_ was determined, assuming LMG do not exhibit mass loss.

## Data Availability

The original contributions presented in this study are included in the article/Supplementary Materials. Further inquiries can be directed to the corresponding author.

## Supplementary Materials

The following supporting information can be downloaded at https://www.mdpi.com/article/10.3390/polym17192679/s1. References [41,42,43,44,45] are cited in the Supplementary Materials.
