# Supplementary material for: Phosphate Low-Melting Glasses as Synergist in Flame-Retardant Cable Sheath Composition: Performance and Mode of Action
Source: Polymers (Basel). 2025 Oct 3;17(19):2679. doi: 10.3390/polym17192679 (PMC12526736; doi:10.3390/polym17192679)
Supplement: Supplementary file 1 [file polymers-17-02679-s001.zip › polymers-3874104-supplementary.pdf]

## Supplementary materials

### SM1

In order to evaluate the mismatch between HRR curves, a relative error index $\varepsilon$ was calculated. This latter represents the surface area between a given curve n and a reference curve, divided by the surface area of the reference curve (THR). Values of mismatch $\varepsilon$ are presented in table below. Up to 25%, it can be estimated that the shapes of the curves are relatively close.

$$\varepsilon = \frac{\sum_{t=0}^{t=t_f} |HRR_n(t) - HRR_{ref}(t)| \times \Delta t}{\sum_{t=0}^{t=t_f} HRR_{ref}(t) \times \Delta t}$$

**Table SM1 :** relative error index $\varepsilon$ for the different composition

| Formulations | ε (%) |
|---|---|
| PE-EVA/ATH | 0.9 |
| PE-EVA/ATH/LMG1 | 2.4 |
| PE-EVA/ATH/LMG2 | 5.3 |
| PE-EVA/ATH/LMG3 | 1.2 |

### SM2

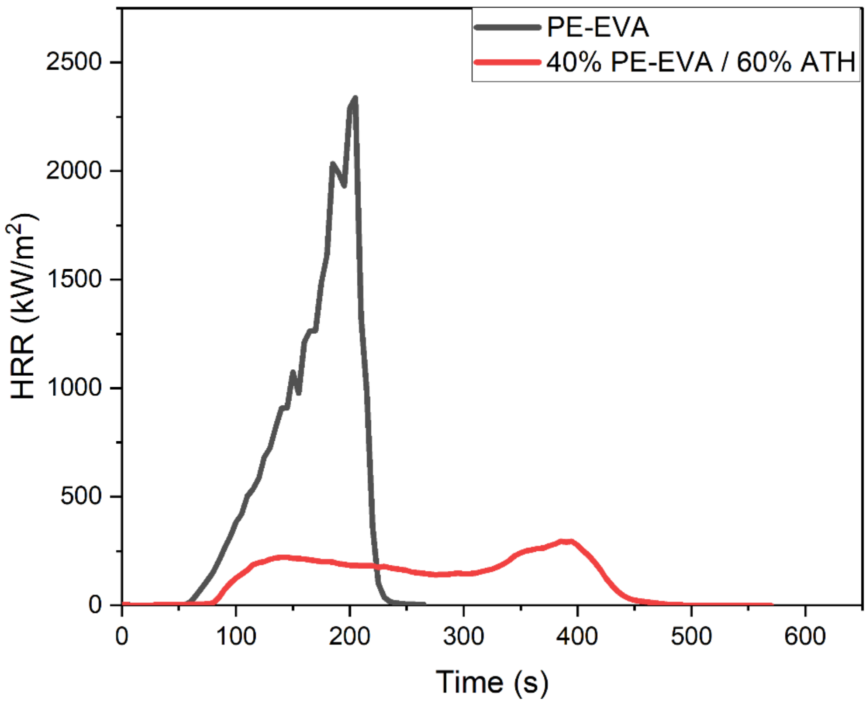


**Figure SM2.** HRR curves for PE-EVA and PE-EVA/ATH (40/60).

## SM3

**Table SM3:** Performance of PE-EVA/ATH/LMG3 compared to literature

| Formulation | Irradiance | pHRR (kW/m²) | pHRR reduction (%)* | Reference |
|---|---|---|---|---|
| 40%EVA- 55%MDH-5%MMT** | 50 | 220 | 40.5 | [14] |
| 40%EVA-45%MDH-15%Talc | 50 | 225 | 39.2 | [14] |
| 40%EVA-50%MDH-5%MMT-5%Silice | 50 | 168 | 50.0 | [76] |
| 75%EVA-25%Pseudo-boehmite | 50 | 460 | 44.8 | [15] |
| 40%EVA-55%ATH-5%Diatomite | 50 | 145 | 30.3 | [18] |
| 43.5%EVA-47%ATH-9.5%Melamine Borate | 35 | 117 | 19.9 | [77] |
| 41%EVA-54%ATH-5%MMT | 50 | 152 | 31.2 | [78] |
| 60%EVA-37%ATH-3%EG** | 50 | 300 | 61.4 | [79] |
| 32%EVA-63%ATH-5%NC** | 50 | 160 | 5.9 | [80] |
| 40%PE-EVA-50%ATH-10%LMG3 | 50 | 142 | 51.9 | Present sudy |

* pHRR reduction was calculated by dividing the pHRR of the composition containing synergist by the pHRR of the corresponding composition containing only ATH or MDH

** MDH : magnesium dihydroxide, MMT : montmorillonite, NC : nanoclay, EG : expandable graphite
